# ADDED VALUE OF TIMING DATA FOR THE ESTIMATION OF COGNITIVE FUNCTIONING IN SURVEY RESEARCH

**DOI:** 10.1093/geroni/igae098.1457

**Published:** 2024-12-31

**Authors:** Emma Nichols, Michael Markot, Alden Gross, Richard Jones, Stefan Schneider, Jinkook Lee

**Affiliations:** University of Southern California, Los Angeles, California, United States; University of Southern California, Los Angeles, California, United States; Johns Hopkins Bloomberg School of Public Health, Baltimore, Maryland, United States; Brown University, Providence, Rhode Island, United States; University of Southern California, Los Angeles, California, United States; University of Southern California, Los Angeles, California, United States

## Abstract

The use of speeded tests to assess cognition supports the notion that response times on cognitive tests contain meaningful information about cognitive functioning. Although information on the time spent completing cognitive tests is often collected during survey administration, this data is not typically considered when quantifying cognitive functioning in large-scale surveys. We used data from the Longitudinal Aging Study in India – Diagnostic Assessment of Dementia (LASI-DAD) study (N=4,091), to assess the added value of data on the time spent on cognitive tests (items), over and above scores from traditional scoring procedures. We used separate linear regression models for each item (N items=35) to assess associations between cognitive factor scores based on all items and quintile of time spent on specific items, adjusting for age, gender, and the item-specific score. Compared to Quintile 3 (median amount of time), taking longer was significantly (p< 0.05) associated with lower cognitive functioning for 54% (Quintile 5) and 31% (Quintile 4) of items. Responding quickly (Quintile 1) was associated with higher cognitive functioning for 50% of simple items (e.g., what is the year), but was associated with lower cognitive functioning for 53% of items requiring higher-order processing (e.g., digit span test). Results were similar in models controlling for education, interviewer, or language of administration. Overall, data show that information on response times from the administration of cognitive tests may contain important information on cognition not captured in traditional scoring. Future research should assess how this information could be leveraged to improve estimates of cognitive functioning.

